# Discontinuation risk comparison among ‘real-world’ newly anticoagulated atrial fibrillation patients: Apixaban, warfarin, dabigatran, or rivaroxaban

**DOI:** 10.1371/journal.pone.0195950

**Published:** 2018-04-30

**Authors:** Gregory Y. H. Lip, Xianying Pan, Shital Kamble, Hugh Kawabata, Jack Mardekian, Cristina Masseria, Hemant Phatak

**Affiliations:** 1 University of Birmingham Institute of Cardiovascular Sciences, City Hospital, Birmingham, United Kingdom; 2 Aalborg Thrombosis Research Unit, Department of Clinical Medicine, Aalborg University, Aalborg, Denmark; 3 Center for Observational Research and Data Science, Bristol-Myers Squibb Company (BMS), Princeton, New Jersey, United States of America; 4 Worldwide Health Economics and Outcomes Research, Bristol-Myers Squibb Company (BMS), Princeton, New Jersey, United States of America; 5 Outcomes & Evidence, Patient & Health Impact, Pfizer Inc., New York, New York, United States of America; Institut d’Investigacions Biomediques de Barcelona, SPAIN

## Abstract

Discontinuation of oral anticoagulants may expose non-valvular atrial fibrillation (NVAF) patients to an increased risk of stroke. This study describes the real-world discontinuation rates and compared the risk of drug discontinuation among NVAF patients initiating apixaban, warfarin, dabigatran, or rivaroxaban. This retrospective cohort study evaluated newly-anticoagulated NVAF patients in the MarketScan^®^ data population from 01/01/2012 through 12/31/2014. Discontinuation was defined as a lack of subsequent prescription of the index drug within 30 days after the last supply day of the last prescription. A Cox model was used to estimate the hazard ratio (HR) of discontinuation, adjusted for age, sex, and comorbidities. Among 45,361 eligible NVAF patients, 15,461 (34.1%) initiated warfarin; 7,438 (16.4%) apixaban; 4,661 (10.3%) dabigatran; and 17,801 (39.2%) initiated rivaroxaban treatment. Compared to warfarin, patients who initiated dabigatran (adjusted HR [aHR]: 0.84, 95% confidence interval [CI]: 0.80–0.87, P<0.001), rivaroxaban (aHR: 0.70, 95% CI: 0.68–0.73, P<0.001), or apixaban (aHR: 0.57, 95% CI: 0.55–0.60, P<0.001) were 16%, 30%, and 43% less likely to discontinue treatment, respectively. When compared to apixaban, patients who initiated dabigatran (aHR: 1.46, 95% CI: 1.38–1.54, P<0.001) or rivaroxaban (aHR: 1.23, 95% CI: 1.17–1.28, P<0.001) were more likely to discontinue treatment. Among newly-anticoagulated NVAF patients in the real-world setting, initiation on rivaroxaban, dabigatran, or apixaban was associated with a significantly lower risk of discontinuation compared to warfarin. When compared to apixaban, patients who initiated treatment with warfarin, dabigatran, or rivaroxaban were more likely to discontinue treatment.

## Introduction

While warfarin is highly effective for preventing stroke in patients with atrial fibrillation (AF), there is significant inter- and intra-patient variability in dose requirements, thus necessitating regular anticoagulation monitoring. Warfarin is also associated with many diet and drug interactions and can be associated with a significant rate of major bleeding, particularly intracranial hemorrhage [1,2]. Given these difficulties, many at-risk AF patients do not receive warfarin or receive an inadequate dose, and often discontinue therapy [1,2]. As a result, approximately 30–50% of patients have been undertreated with either suboptimal warfarin treatment, aspirin, or no anticoagulation [3]. In recent years, 4 non-Vitamin K antagonist oral anticoagulants (NOACs), including dabigatran, rivaroxaban, apixaban, and edoxaban, have been approved in the United States for stroke prevention in NVAF patients. The NOACs have advantages over warfarin in that there is no need for regular International Normalized Ratio (INR) monitoring, and there are fewer drug and food interactions. In clinical trials assessing warfarin, dabigatran, rivaroxaban, apixaban, and edoxaban, all have been shown to be safe and effective [4–7]. Among all NOACs approved for non-valvular AF (NVAF), apixaban is the only drug that has shown, in its respective clinical trial(s), to have a significantly lower discontinuation rate than warfarin (or aspirin) possibly due to its significantly better safety and tolerability profile [6,8,9]. Due to the moderate half-life of NOACs, it is important for patients to continue to take medications daily, as indicated. For both warfarin and NOACs, drug discontinuation and missing doses (ie, lower adherence) may expose AF patients to an increased risk of stroke. Consequently, studies of discontinuation and adherence associated with NOACs are important in understanding treatment patterns and associated gaps [10,11,12]. It would be important to investigate discontinuation rates and time to discontinuation associated with various NOACs to understand the ability of patients to continue long-term NOAC use in real-world settings. The objective of this study was to describe the ‘real-world’ discontinuation rates and compare the risk of drug discontinuation among patients initiating apixaban, dabigatran, rivaroxaban, and warfarin. For the purposes of this analysis, there were insufficient real-world data available for edoxaban in the United States.

## Methods

A retrospective cohort study was conducted using the Truven MarketScan^®^ Commercial Claims & Encounters and Medicare Supplemental & Coordination of Benefits database (January 2012 through December 2014) to evaluate the discontinuation rates among AF patients who initiated apixaban, warfarin, dabigatran, or rivaroxaban treatment [13]. The database includes health insurance claims for over 60 million employees, spouses, retirees, and their dependents, enrolled in employer-sponsored commercial and Medicare advantage plans. The geographical distribution is approximately the same as the US population distribution. The database includes fully-integrated health information, including inpatient and outpatient health care resource utilization and detailed drug information. The pharmaceutical claims file includes complete records of prescriptions, including mail-order or card program prescription drug claims [13].

**Fig 1:**
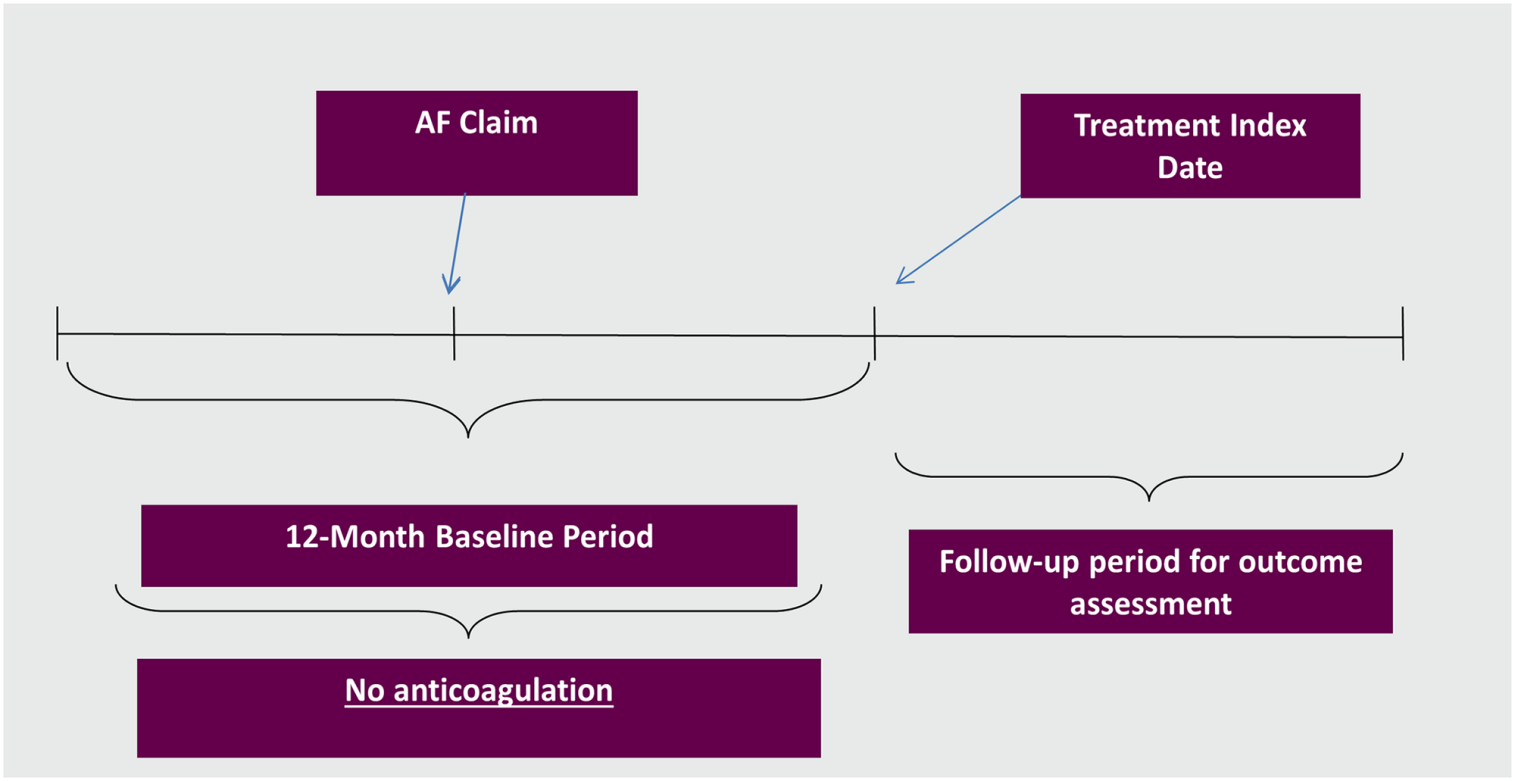
Study period depiction. Study period for patients initiating apixaban, dabigatran, rivaroxaban, and warfarin. AF: atrial fibrillation.

NVAF patients aged ≥18 years (identified based on presence of at least 1 claim for a primary or secondary diagnosis of AF in the inpatient or outpatient setting using ICD-9-CM code 427.31 or 472.32) with a 1-year baseline period with continuous health plan enrollment were included if they were newly prescribed oral anticoagulants from January 1, 2013 through December 31, 2014 [14]. The ICD-9 codes have been validated for identifying AF patients with a median positive predictive value of 89% [14]. In other words, a contemporary cohort of NOAC and warfarin initiators without prior oral anticoagulant treatment during the 1-year baseline period was selected for this study. The index drug was defined as the first anticoagulation treatment prescribed to patients included in the study. The index date was defined as the first index drug prescription date, after NVAF diagnosis. The index date is depicted in Fig 1.

**Fig 2:**
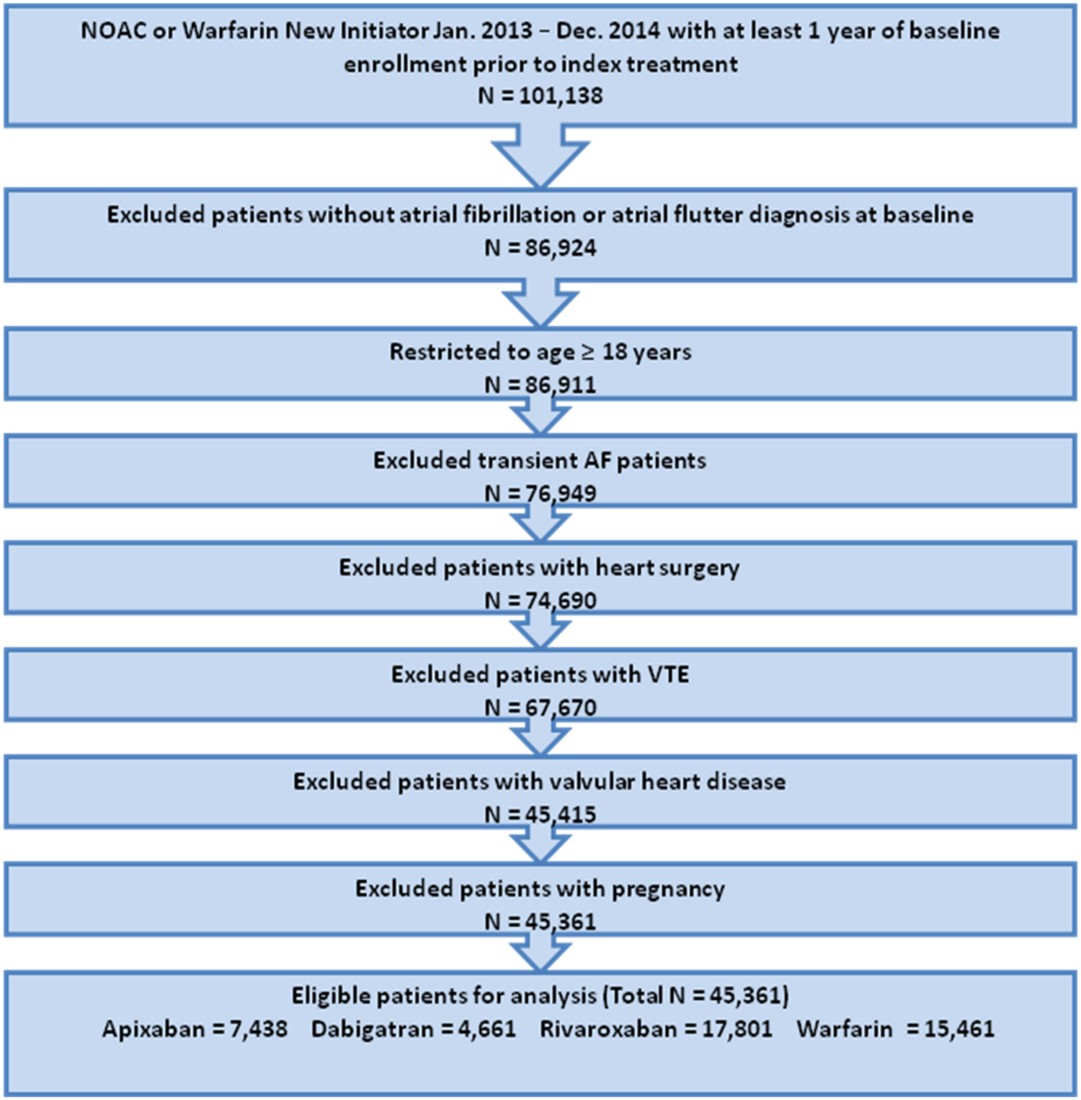
Patient selection criteria. Study population flow chart with inclusion and exclusion criteria used to select 45,361 patients. NOAC: non-vitamin K antagonist oral anticoagulant; VTE: venous thromboembolism.

Patients with claims including ICD-9-CM diagnosis codes for transient AF (pericarditis, hyperthyroidism, thyrotoxicity), valvular heart disease, venous thromboembolism, cardiac surgery, or pregnancy during the baseline period (any time prior to or on the index date) were excluded (Fig 2; S1 Table).

Discontinuation was defined as a gap in therapy when a subsequent prescription for the index treatment occurred >30 days, plus the days supply of the previous prescription [12]. Patients who did not discontinue treatment were followed until the date of a switch to another anticoagulant, end of the study period, inpatient death, or interruption in continuous health plan enrollment, whichever occurred earliest. A sensitivity analysis was completed by changing the discontinuation gap to 60 days and 90 days.

A second sensitivity analysis was also performed. Due to varying mean length of follow-up across treatment cohorts, only patients who had at least 100 days of follow-up were included, in order to assess the robustness of the estimated risk of discontinuation.

In the study population, categorical and continuous variables were compared across treatments using the Pearson chi-square test and the Wilcoxon rank-sum test, respectively. The proportions of patients discontinuing therapy during the study follow-up period were computed as the number of patients who discontinued treatment divided by the total number of patients initiating the treatment within the study period. The cumulative incidence of discontinuation was presented using Kaplan–Meier curves. A Cox proportional hazards model was used to estimate the hazard ratios (HRs) of discontinuation for each drug compared to apixaban, adjusted for a pre-specified set of baseline demographic and clinical factors including age, sex, embolic or primary ischemic stroke, dyspepsia or stomach discomfort, congestive heart failure, coronary artery disease, diabetes mellitus, hypertension, renal disease, myocardial infarction, history of stroke, or transient ischemic attack, and history of bleeding (S1 Table).

As warfarin is the standard of care, we have analyzed and presented the primary results using warfarin as a reference comparator. Lower discontinuation rates were observed for NOACs in real-world studies and clinical trials; therefore, we also analyzed apixaban as a reference cohort to understand the discontinuation risk associated with NOACs and warfarin in the real-word setting. All analyses were performed with SAS System version 9.2. An *a priori* significance level of 0.05 was used for the purposes of these analyses.

## Results

Among 45,361 patients, 15,461 (34.1%) initiated warfarin treatment with a mean (SD) follow-up of 164 (±162) days; 7,438 (16.4%) initiated apixaban with a mean follow-up of 148 (±138) days; 4,661 (10.3%) initiated dabigatran with a mean follow-up of 177 (±179) days; and 17,801 (39.2%) patients initiated rivaroxaban with a mean follow-up of 176 (±171) days (Table 1). Approximately 60% of patients in each treatment group were male. The mean age of warfarin, apixaban, dabigatran, and rivaroxaban patients was 71.7±12.0, 68.5±12.4, 66.5±12.4, and 67.1±12.3 years, respectively. Patients initiating warfarin were older and were at higher risk in terms of the CHA_2_DS_2_-VASc score (3.2±1.7) and had higher mean Charlson comorbidity index (CCI) scores of 2.3±2.3 (Table 1).

**Table 1:** Baseline patient characteristics and treatment follow-up period.

	Apixaban (n = 7,438)		Dabigatran (n = 4,661)		Rivaroxaban (n = 17,801)		Warfarin (n = 15,461)	
	N/Mean	%/SD	N/Mean	%/SD	N/Mean	%/SD	N/Mean	%/SD
Age	68.5	12.4	66.5	12.4	67.1	12.3	71.7	12.1
18–64	3111	41.8	2234	47.9	8245	46.3	4845	31.3
65–74	1805	24.23	1096	23.5	4208	23.6	3595	23.3
75+	2522	33.9	1331	28.6	5348	30.0	7021	45.4
Sex								
Male	4566	61.4	3028	65.0	11310	63.5	9254	59.9
Female	2872	38.6	1633	35.0	6491	36.5	6207	40.2
Embolic or Primary Ischemic Stroke	523	7.0	259	5.6	1056	5.9	1551	10.0
Dyspepsia or Stomach Discomfort	1082	14.6	564	12.1	2548	14.3	2470	16.0
Congestive Heart Failure	1454	19.6	873	18.7	3293	18.5	4021	26.0
Coronary Artery Disease	2410	32.4	1283	27.5	5112	28.7	5305	34.3
Diabetes	2108	28.3	1269	27.2	4802	27.0	4987	32.3
Hypertension	5585	75.1	3253	69.8	12690	71.3	11334	73.3
Renal Disease	638	8.6	333	7.1	1398	7.9	2242	14.5
Myocardial Infarction	469	6.3	251	5.4	1089	6.1	1378	8.9
History of Stroke or TIA	754	10.1	413	8.9	1574	8.8	2061	13.3
History of Bleeding	1012	13.6	536	11.5	2421	13.6	2800	18.1
CHA_2_DS_2_-VASc Score*	2.8	1.6	2.6	1.7	2.6	1.7	3.2	1.7
0	514	6.9	485	10.4	1650	9.3	766	5.0
1	1222	16.4	908	19.5	3401	19.1	1809	11.7
2	1644	22.1	1027	22.0	3884	21.8	2750	17.8
≥3	4058	54.6	2241	48.1	8866	49.8	10136	65.6
Charlson Comorbidity Index Score	1.8	2.0	1.6	1.9	1.7	2.0	2.3	2.3
0	2244	30.2	1631	35.0	6117	34.4	3741	24.2
1	1899	25.5	1201	25.8	4468	25.1	3434	22.2
2	1270	17.1	729	15.6	2721	15.3	2499	16.2
≥3	2025	27.2	1100	23.6	4495	25.3	5787	37.4
Treatment Follow-up (in days)	148	138	177	178	175	171	164	162
Median	95	-	100	-	111	-	101	-
IQR	36–215	-	30–255	-	35–261	-	40–233	-

SD: Standard Deviation; TIA: Transient Ischemic Attack; IQR: Interquartile Range.

*The CHA_2_DS_2_-VASc score was calculated as the sum of points associated with each of the following attributes: congestive heart failure (1 point), hypertension (1 point), age ≥75 (2 points), diabetes (1 point), prior stroke or TIA (2 points), vascular disease (1 point), age 65–74 (1 point), female (1 point).

**Fig 3:**
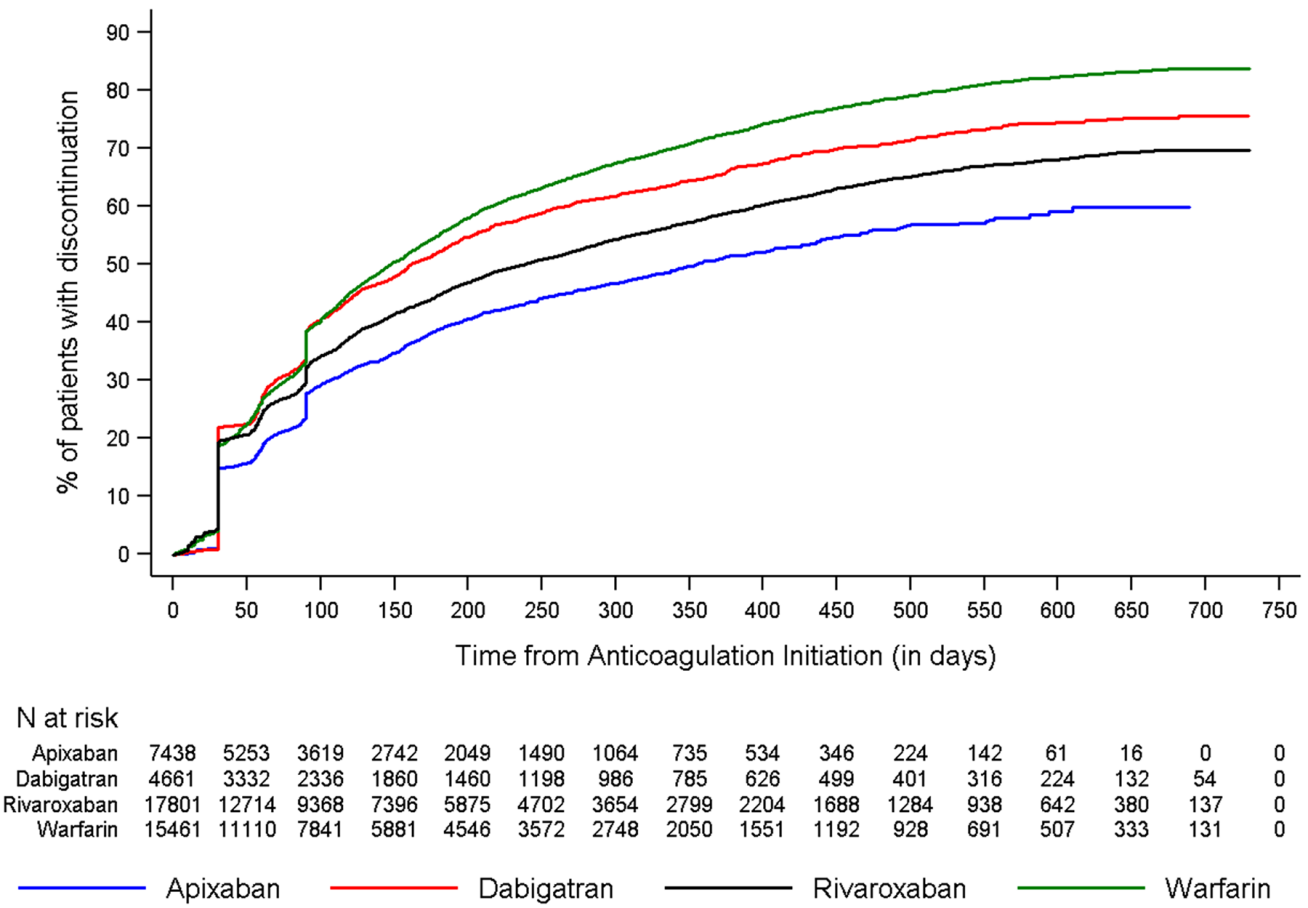
Cumulative incidence of discontinuation among newly anticoagulated non-valvular atrial fibrillation patients. (Upper panel) Cumulative incidence of discontinuation during the follow-up period. The unadjusted cumulative incidence of discontinuation was lower among patients initiated on apixaban compared to patients inititated on other oral anticoagulants. (Lower panel) The number of patients at risk for discontinuation at varying points during the follow-up.

Fig 3 shows the discontinuation rates of all drugs across the study period. The cumulative incidence of discontinuation at one-year was 50.5%, 64.7%, 57.8%, and 71.6% for apixaban, dabigatran, rivaroxaban, and warfarin patients, respectively. When the 60- and 90-day gap was used, the rates of discontinuation were lower but the trend was consistent (S2 Table, S2 and S3 Figs). Across the four cohorts, around 3–10% of patients switched from index OAC to another OAC during the follow-up period.

When *compared to warfarin*, patients who initiated dabigatran (adjusted HR [aHR]: 0.84, 95% confidence interval [CI]: 0.80–0.87), rivaroxaban (aHR: 0.70, 95% CI: 0.68–0.73, P<0.001), or apixaban (aHR: 0.57, 95% CI: 0.55–0.60, P<0.001) were 16%, 30%, and 43% less likely to discontinue treatment, respectively.

When *compared to apixaban*, patients who initiated warfarin (aHR: 1.74, 95% CI: 1.67–1.82, P<0.001), dabigatran (aHR: 1.46, 95% CI: 1.38–1.54, P<0.001), and rivaroxaban (aHR: 1.23, 95% CI: 1.17–1.28, P<0.001) were significantly more likely to discontinue treatment, after adjusting for baseline characteristics (Table 2 and Fig 3).

**Table 2:** Adjusted hazard ratios of discontinuation.

	Hazard Ratio*	**Hazard Ratio 95% Confidence Limits**		**P-value**	Hazard Ratio*	**Hazard Ratio 95% Confidence Limits**		**P-value**
Warfarin	1.74	1.67	1.82	<0.001	1.00 Reference			
Dabigatran	1.46	1.38	1.54	<0.001	0.84	0.80	0.87	<0.001
Rivaroxaban	1.23	1.17	1.28	<0.001	0.70	0.68	0.73	<0.001
Apixaban	1.00 Reference				0.57	0.55	0.60	<0.001
*Covariates Included in Both Models Have the Same Estimates as Shown Below*								
			Hazard Ratio*		**Hazard Ratio 95% Confidence Limits**		**P-value**	
Age (75+ as a reference category)								
18–64			1.34		1.30	1.38	<0.001	
65–74			0.99		0.96	1.03	0.554	
Male			1.02		0.99	1.05	0.226	
Embolic or Primary Ischemic Stroke			0.96		0.88	1.05	0.394	
Dyspepsia or Stomach Discomfort			1.10		1.06	1.14	<0.001	
Congestive Heart Failure			0.96		0.93	0.99	0.007	
Coronary Artery Disease			1.01		0.98	1.04	0.557	
Diabetes			0.89		0.87	0.92	<0.001	
Hypertension			0.89		0.86	0.91	<0.001	
Renal Disease			1.04		0.99	1.08	0.116	
Myocardial Infarction			1.05		0.99	1.11	0.093	
History of Stroke or Transient Ischemic Attack			0.85		0.79	0.92	<0.001	
History of Bleeding			1.12		1.08	1.16	<0.001	

*Adjusted hazard ratios were estimated from a Cox proportional hazards model with factors for age, sex, embolic or primary ischemic stroke, dyspepsia or stomach discomfort, congestive heart failure, coronary artery disease, diabetes, hypertension, renal disease, myocardial infarction, history of stroke or transient ischemic attack, and history of bleeding.

For sensitivity analysis, discontinuation was assessed among patients with at least 100 days of follow-up, given the varied mean follow-up length across treatment cohorts in the main analysis. This reduced the effective sample size to 13,294 patients (35.50%) initiating warfarin, 5,353 (14.29%) apixaban, 4,161 (11.11%) dabigatran, and 14,644 (39.10%) rivaroxaban.

When *compared to warfarin*, patients who initiated dabigatran (aHR: 0.89, 95% CI: 0.85–0.92, P<0.001), rivaroxaban (aHR: 0.72, 95% CI: 0.70–0.74, P<0.001), or apixaban (aHR: 0.61, 95% CI: 0.58–0.64, P<0.001) were less likely to discontinue treatment (S3 and S4 Tables and Fig 3). When *compared to apixaban*, patients who initiated warfarin (aHR: 1.64, 95% CI: 1.57–1.71, P<0.001), dabigatran (aHR: 1.45, 95% CI: 1.37–1.53, P<0.001), or rivaroxaban (aHR: 1.18, 95% CI: 1.12–1.23, P<0.001) were more likely to discontinue treatment (S1 and S3 Tables; Fig 1).

## Discussion

When compared to those who initiated warfarin, patients who initiated dabigatran, rivaroxaban, or apixaban were less likely to discontinue treatment after adjusting for baseline characteristics. Further, when compared to apixaban, patients who initiated treatment with warfarin, dabigatran, or rivaroxaban were more likely to discontinue treatment.

This study used real-world claims data from the US population to provide critical insights regarding the risk of discontinuation in an adult NVAF population who initiated anticoagulant treatment with apixaban, warfarin, rivaroxaban, or dabigatran therapy. Importantly, these ‘real-world’ results are consistent with the clinical trial findings for the respective NOACs and warfarin. In the ARISTOTLE trial, for example, fewer patients in the apixaban group (25.3%) compared to the warfarin group (27.5%) discontinued the study drug before the end of the study (p = 0.001) [6]. In the ROCKET-AF trial, the proportion of patients who permanently stopped their assigned therapy before an end-point event and before the termination date was 23.7% in the rivaroxaban group and 22.2% in the warfarin group [5]. In the RE-LY trial, the rates of discontinuation for 110 mg of dabigatran, 150 mg of dabigatran, and warfarin were 14.5%, 15.5%, and 10.2%, respectively, at 1 year and 20.7%, 21.2%, and 16.6%, respectively, at 2 years [4].

The robustness of these findings through sensitivity analysis was also assessed, which largely concurred with our primary findings by demonstrating a lower risk of discontinuation with NOAC initiation as compared to warfarin, and a higher risk of discontinuation with initiation on other anticoagulants as compared to apixaban.

Our results also confirm findings from recent ‘real-world’ studies. A long-term study of the different NOACs in clinical practice showed that discontinuation rates were lower for apixaban (10%) compared to dabigatran (30%) and rivaroxaban (25%; p<0.001 for both) [15]. Another study of NVAF patients who initiated oral anticoagulants showed significantly higher 1-year persistence rates with apixaban (86%) and warfarin (85%) than with dabigatran (74%) or rivaroxaban (77%) [16]. Furthermore, in a propensity score-matched analysis, patients who initiated dabigatran (63%) had higher persistence rates at 1 year compared to patients who initiated warfarin (39%) [12].

Discontinuation of anticoagulation or poor adherence to thromboprophylaxis is not inconsequential. Indeed, previous studies have found that warfarin discontinuation is associated with increased risk of ischemic stroke. For example, Ewen, et al. [17] found that patients with 1 or 2 or more warfarin interruptions (defined as prescription gaps over 45 days) had higher stroke incidence than those without warfarin interruption (relative risk: 2.29; 95% CI: 1.29–4.07). Deitelzweig, et al. also found that stroke risk was higher with warfarin discontinuation than during continuous warfarin therapy (HR: 1.60; 95% CI: 1.35–1.90) [18]. Similarly, in a study by Spivey et al., discontinuation versus persistent use of warfarin was associated with increased risk of ischemic stroke (HR: 2.04; 95% CI: 1.47–2.84) and ischemic stroke or TIA (HR: 1.50; 95% CI: 1.20–1.87) [19].

The anticoagulant effect of NOACs decreases rapidly; therefore, poor adherence and treatment discontinuation may diminish the benefit of NOAC treatment [20]. A higher risk of thromboembolism was noted at the end of the double-blind trials during the transition from rivaroxaban to open-label warfarin [21]. A recent analysis from the ROCKET-AF trial showed an increased risk of stroke and non-central nervous system embolism among rivaroxaban-treated versus warfarin-treated AF patients who temporarily or permanently discontinued anticoagulation (HR: 1.50, 95% CI: 1.05–2.15) [21]. Furthermore, a longitudinal outcomes study using a national cohort from the Veterans Health Administration showed that lower adherence to dabigatran was associated with an increased risk of combined all-cause mortality and stroke (HR: 1.13, 95% CI: 1.07–1.19 per 10% decrease in proportion of days covered) [22]. However, several ‘real-world’ studies have demonstrated reduced or similar ischemic stroke risk among patients treated with NOACs compared with warfarin, suggesting adequate adherence to maintain the benefits of NOACs [20].

Our results are also consistent with indirect comparisons that combined data from the RELY, ROCKET-AF, and ARISTOTLE studies in relation to the discontinuation of treatment [23]. While the reasons for discontinuation, causality between any particular attribute, and discontinuation of therapy cannot be determined in this retrospective study, the safety and tolerability profile of a drug likely plays a critical role in patients’ ability to use NOACs continuously. For example, a 2012 study showed the rate of discontinuation of dabigatran was 25.4%, with dyspepsia being the most common reason for discontinuation [24]. Further, a single clinical center cross-sectional study in the United States reported that the most common reasons for discontinuation with dabigatran were adverse reactions and cost [25].

The study’s strengths are that we assessed the real-world risk of discontinuation of treatment among patients initiating warfarin versus dabigatran, rivaroxaban, and apixaban using the comprehensive MarketScan^®^ claims database, which incorporates all medical and pharmacy patient claims and allows for longitudinal analysis of a nationally-representative sample. The medications being studied are relatively new to the market, and this database, encompassing both commercial and Medicare beneficiaries, allows for the selection of a nationally-representative sample for this study [13].

As with any retrospective analysis, researchers are limited to only study associations between variables; additionally, as with any retrospective observational database study, there is a potential for selection bias. We conducted rigorous and thorough multivariate and sensitivity analyses for discontinuation to ensure the robustness of our findings. Baseline comorbidities (eg, presence of renal impairment) were determined by the presence of a diagnosis code in the baseline period and were not based on actual laboratory test result values or clinical assessment. As this study is an analysis of claims data, there is the potential for coding errors or missing data (ie, we must assume that patients do not have a condition if it was not coded). Additionally, refill data may not reflect actual medication use. Discontinuation rates of warfarin, as assessed by a 30-day gap in pharmacy claims, may vary more due to dose adjustments to manage fluctuation in INR values compared to NOACs. As apixaban had entered the market recently when the analysis was performed, patients treated with apixaban tended to have a shorter follow-up than those treated with warfarin.

Further analyses using propensity-matched cohorts as well as large-scale prospective studies may be necessary to understand the reasons for and predictors of discontinuation, and to examine the impact of discontinuation on clinical outcomes.

## Conclusions

Among newly-anticoagulated AF patients in the real-world setting, treatment initiation with rivaroxaban, dabigatran, or apixaban was associated with a significantly lower risk of discontinuation as compared to warfarin. Treatment discontinuation risk was significantly higher among patients initiating dabigatran, rivaroxaban, or warfarin, as compared to those initiating apixaban.

## Supporting information

S1 Fig Cumulative incidence of discontinuation: With a 60-day gap. (TIF)

S2 Fig Cumulative incidence of discontinuation: With a 90-day gap. (TIF)

S3 Fig Cumulative incidence of discontinuation: Minimum 100 days follow-up. (TIF)

S1 Table ICD-9-CM codes for selection criteria and comorbid conditions. (DOCX)

S2 Table Cumulative incidence of discontinuation at 1 year. (DOCX)

S3 Table Patient characteristics: Minimum 100 days follow-up. (DOCX)

S4 Table Adjusted risk of discontinuation: Minimum 100 days follow-up. (DOCX)
