# Supplementary figures and images for: Discontinuation risk comparison among ‘real-world’ newly anticoagulated atrial fibrillation patients: Apixaban, warfarin, dabigatran, or rivaroxaban
Source: PLoS One. 2018 Apr 30;13(4):e0195950. doi: 10.1371/journal.pone.0195950 (PMC5927458; doi:10.1371/journal.pone.0195950)

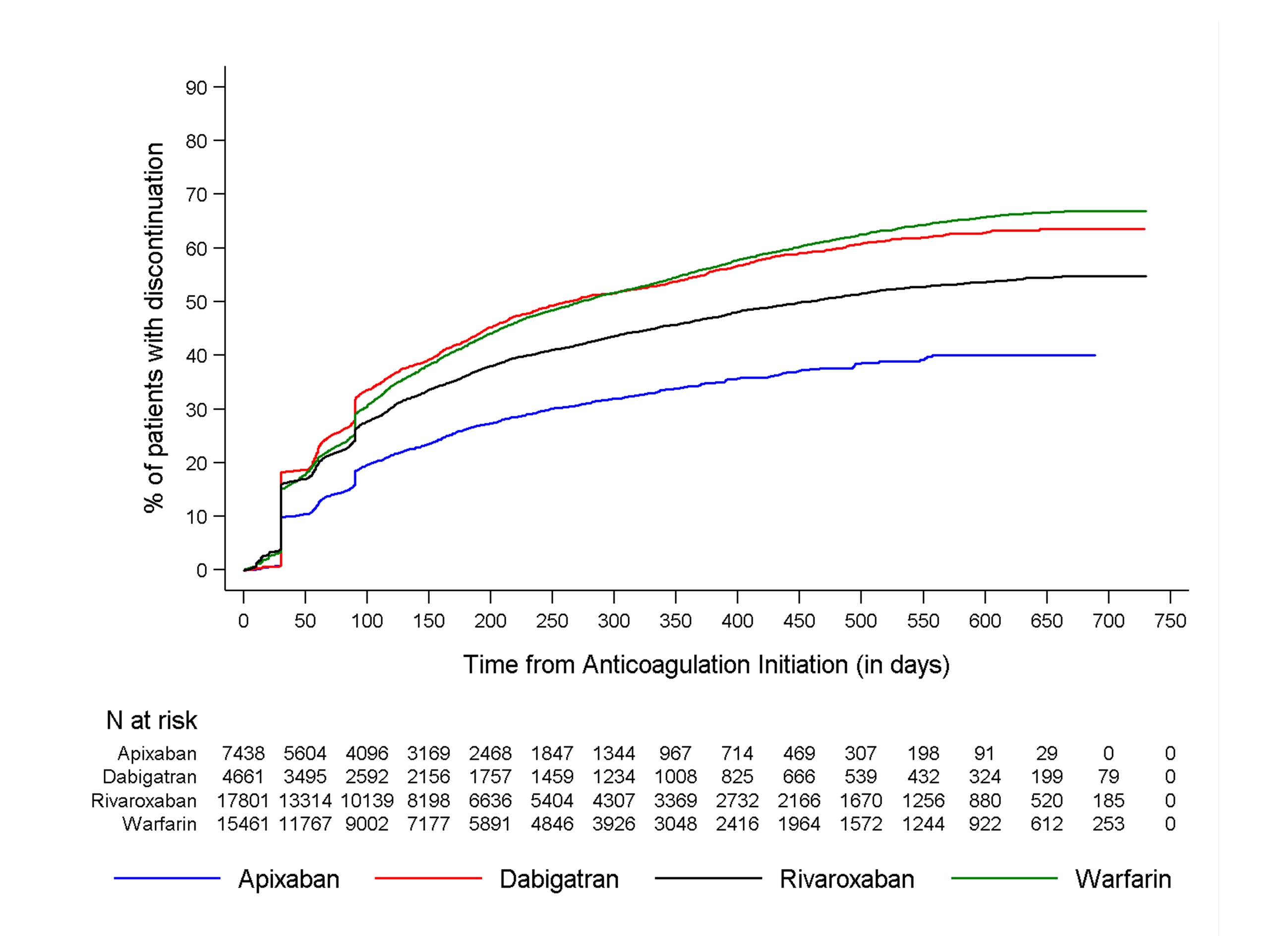

Supplement: S1 Fig — (TIF) [file pone.0195950.s001.tif]

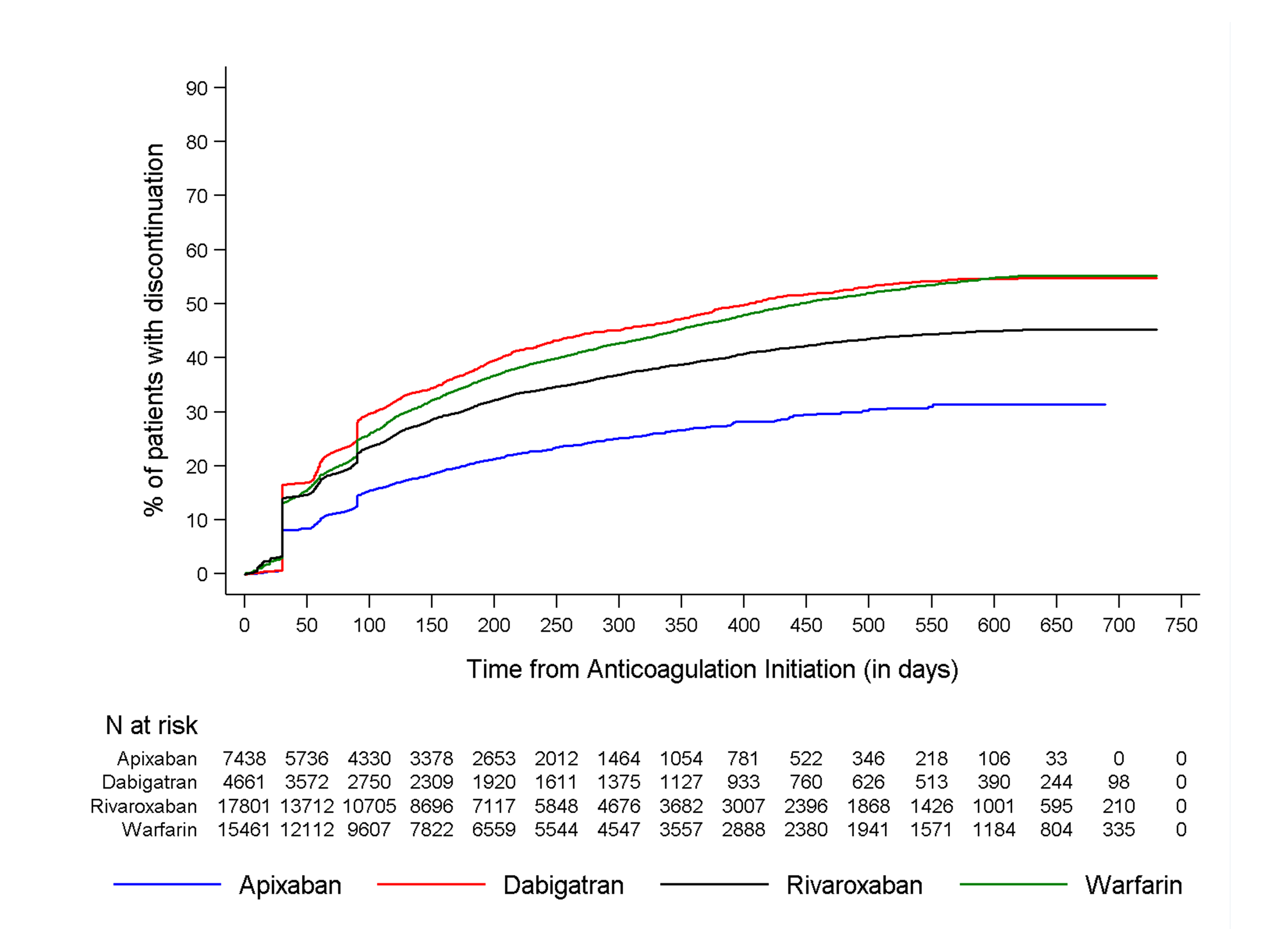

Supplement: S2 Fig — (TIF) [file pone.0195950.s002.tif]

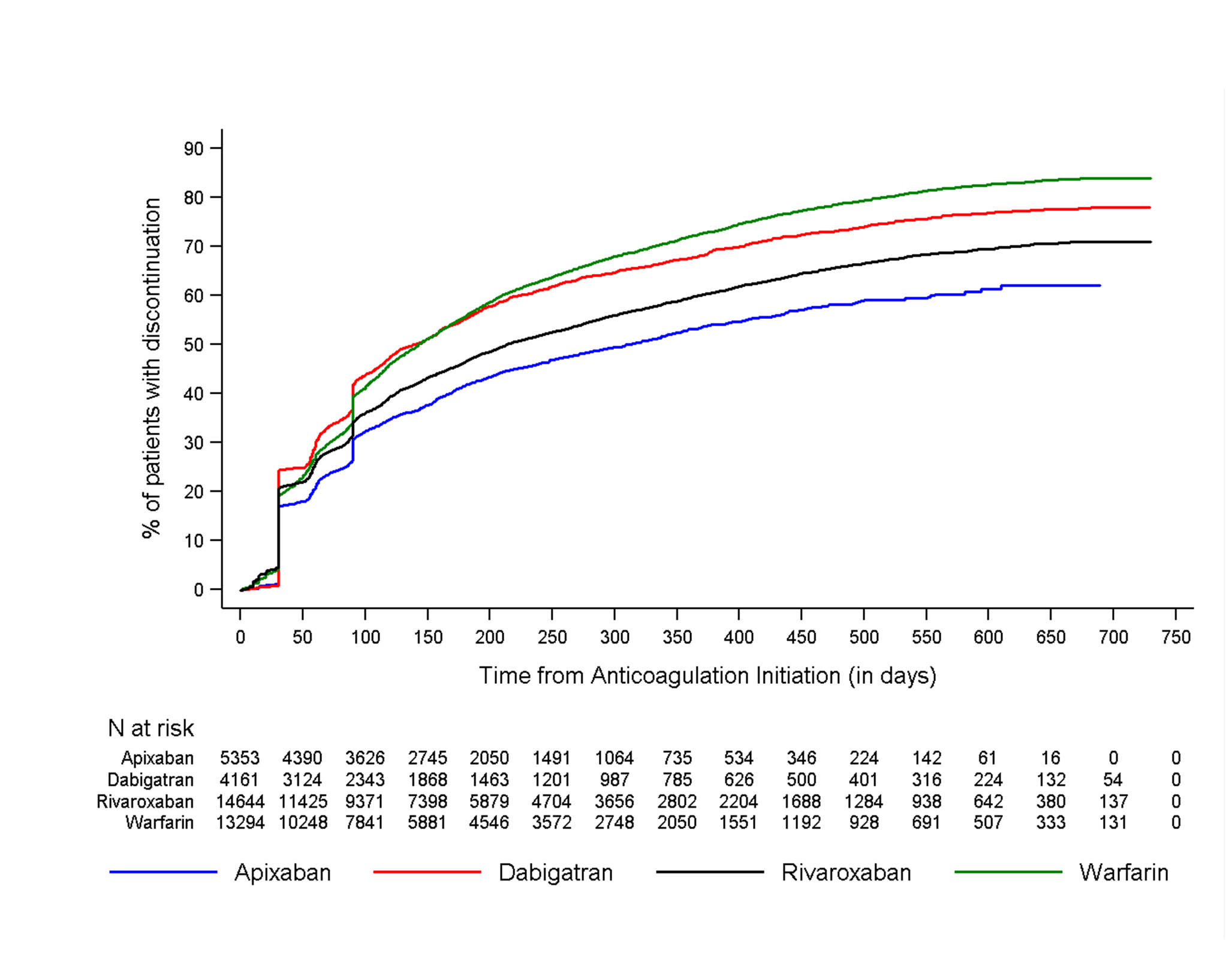

Supplement: S3 Fig — (TIF) [file pone.0195950.s003.tif]
