# Supplementary material for: Discontinuation risk comparison among ‘real-world’ newly anticoagulated atrial fibrillation patients: Apixaban, warfarin, dabigatran, or rivaroxaban
Source: PLoS One. 2018 Apr 30;13(4):e0195950. doi: 10.1371/journal.pone.0195950 (PMC5927458; doi:10.1371/journal.pone.0195950)
Supplement: S1 Table — (DOCX) [file pone.0195950.s004.docx]

**Supplemental Table 1. ICD-9-CM Codes for Selection Criteria and Comorbid Conditions**

| **Diagnosis** | **ICD-9-CM Code^a^** |
| --- | --- |
| Atrial  Fibrillation | 427.31 (atrial fibrillation) or 427.32 (atrial flutter) |
| Valvular Heart Disease | 394.xx, 395.xx, 396.xx, 424.xx, or 746.xx |
| Transient Atrial Fibrillation (heart valve replacement/transplant, pericarditis, hyperthyroidism and thyrotoxicity) | Heart valve replacement/transplant: V422, V433 |
|  | Pericarditis: 006.8, 017.9, 036.41, 074.21, 093.81, 098.83, 115.93, 390, 391, 392.0, 393, 411.0, 420.90, 420.91, 420.99, 423.0, 423.1, 423.2, 423.8, 423.9, 585.9 |
|  | Hyperthyroidism and Thyrotoxicity: 242.0, 242.1, 242.2, 242.3, 242.4, 242.8, 242.9 |
| Pregnancy | Diagnosis codes: 630.xx-649.xx, V22.xx-V23.xx, V28.xx, V61.6x-V61.7x |
|  | Procedure codes: 72.xx-75.xx, 792.3, 796.5 |
|  | HCPCS codes: 59000-59350, 76801-76828, 83661-83664 |
| Congestive Heart Failure | 398.91, 402.x1, 404.x3, 425, 428 |
| Coronary Artery Disease | 410-414 |
| Diabetes Mellitus | 250, 357.2, 362.0, 366.41 |
| Hypertension | 362.11, 401, 402, 403, 404, 405 |
| Renal Disease | 403, 404, 580-586 |
| Myocardial Infarction | 410, 411 |
| Dyspepsia or Stomach Discomfort | 787.1: Heartburn |
|  | 789.0: Abdominal Pain |
|  | 789.4: Abdominal Rigidity |
|  | 789.6: Abdominal Tenderness |
|  | 536.8: Dyspepsia |
| Ischemic Stroke or Transient Ischemic Attack | 433.x1, 434.x1, 435, 436, 437.1, 437.9, 362.34 |
| Baseline Bleeding | 455.2, 455.5, 455.8, 456.0x, 456.20, 530.7x, 530.82, 531.00, 531.01, 531.20, 531.21, 531.40, 531.41, 531.60, 531.61, 532.00, 532.01, 532.20, 532.21, 532.40, 532.41, 532.60, 532.61, 533.00, 533.01, 533.20, 533.21, 533.40, 533.41, 533.60, 533.61, 534.00, 534.01, 534.20, 534.21, 534.40, 534.41, 534.60, 534.61, 535.01, 535.11, 535.21, 535.31, 535.41, 535.51, 535.61, 537.83, 537.84, 562.02, 562.03, 562.12, 562.13, 568.81, 569.3x or 569.85, 569.86, 578.0x, 578.1x, 578.9x  430.xx, 431.xx, 432.0x, 432.1x, 432.9x, , 852.xx, 853.0x,  078.6x, 246.3x, 285.1, 286.5, 388.69, 360.43, 362.43, 362.81, 363.61, 363.62, 363.72, 364.41, 372.72, 374.81, 376.32, 377.42, 379.23, 423.0x, 459.0x, 593.81, 596.7x, 599.7x, 602.1x, 620.1x, 621.4x, 626.2x, 626.5x, 626.7x, 626.8x, 626.9x, 640.xx, 641.9x, 666.1x, 719.1x, 782.7x, 784.7x, 784.8x, 786.3x, 790.01, 958.2x, 997.02, 998.11 |

**^a^** Inpatient and outpatient claims were used
