# Supplementary material for: Discontinuation risk comparison among ‘real-world’ newly anticoagulated atrial fibrillation patients: Apixaban, warfarin, dabigatran, or rivaroxaban
Source: PLoS One. 2018 Apr 30;13(4):e0195950. doi: 10.1371/journal.pone.0195950 (PMC5927458; doi:10.1371/journal.pone.0195950)
Supplement: S2 Table — (DOCX) [file pone.0195950.s005.docx]

**Supplemental Table 2. Patient Characteristics: Minimum 100 days Follow-up**

|  | **Apixaban** | | **Dabigatran** | | **Rivaroxaban** | | **Warfarin** | |
| --- | --- | --- | --- | --- | --- | --- | --- | --- |
|  | **N** | **%** | **N** | **%** | **N** | **%** | **N** | **%** |
| Age | 68.6 | 12.3 | 66.5 | 12.3 | 67.0 | 12.3 | 71.8 | 23.0 |
| 18-64 | 2225 | 41.6 | 1989 | 47.8 | 6795 | 46.4 | 4079 | 30.7 |
| 65-74 | 1284 | 234.0 | 984 | 23.7 | 3439 | 23.5 | 3105 | 23.4 |
| 75+ | 1844 | 34.5 | 1188 | 28.6 | 4410 | 30.1 | 6110 | 46.0 |
| Sex |  |  |  |  |  |  |  |  |
| Male | 3304 | 61.7 | 2689 | 64.6 | 9334 | 63.7 | 7933 | 59.7 |
| Female | 2049 | 38.3 | 1472 | 35.4 | 5310 | 36.2 | 5361 | 40.3 |
| Dyspepsia or Stomach Discomfort | 771 | 14.4 | 502 | 12.1 | 2076 | 14.2 | 2100 | 15.8 |
| Congestive Heart Failure | 1045 | 19.5 | 776 | 18.7 | 2649 | 18.1 | 3410 | 25.7 |
| Coronary Artery Disease | 1701 | 31.7 | 1125 | 27.0 | 4207 | 28.7 | 4539 | 34.1 |
| Diabetes | 1496 | 28.0 | 1108 | 26.6 | 3900 | 26.6 | 4252 | 32.0 |
| Hypertension | 3990 | 74.5 | 2892 | 69.5 | 10345 | 70.6 | 9697 | 72.9 |
| Renal Disease | 433 | 8.1 | 289 | 7.0 | 1111 | 7.6 | 1868 | 14.1 |
| Myocardial Infarction | 326 | 6.1 | 218 | 5.2 | 876 | 6.0 | 1164 | 8.8 |
| History of Stroke or TIA | 522 | 9.8 | 358 | 8.6 | 1287 | 8.8 | 1751 | 13.2 |
| History of Bleeding | 710 | 13.3 | 456 | 11.0 | 1991 | 13.6 | 2406 | 18.1 |

TIA: Transient Ischemic Attack
