# Supplementary material for: Discontinuation risk comparison among ‘real-world’ newly anticoagulated atrial fibrillation patients: Apixaban, warfarin, dabigatran, or rivaroxaban
Source: PLoS One. 2018 Apr 30;13(4):e0195950. doi: 10.1371/journal.pone.0195950 (PMC5927458; doi:10.1371/journal.pone.0195950)
Supplement: S4 Table — (DOCX) [file pone.0195950.s007.docx]

**Supplemental Table 4. Adjusted Risk of Discontinuation: Minimum 100 days Follow-up**

|  | **Hazard Ratio*** | **Hazard Ratio 95% Confidence Limits** | | | **P-value** | **Hazard Ratio*** | | **Hazard Ratio 95% Confidence Limits** | | | | **P-value** |
| --- | --- | --- | --- | --- | --- | --- | --- | --- | --- | --- | --- | --- |
| Warfarin | 1.64 | 1.57 | 1.71 | | <0.001 | Ref | |  | |  | |  |
| Dabigatran | 1.45 | 1.37 | 1.53 | | <0.001 | 0.89 | | 0.85 | | 0.92 | | <0.001 |
| Rivaroxaban | 1.18 | 1.12 | 1.23 | | <0.001 | 0.72 | | 0.70 | | 0.74 | | <0.001 |
| Apixaban | Ref |  |  | |  | 0.61 | | 0.58 | | 0.64 | | <0.001 |
| *Covariates Included in Both Models Have the Same Estimates as Shown Below* | | | | | | | | | | | | |
|  | | | | **Hazard Ratio*** | | | **Hazard Ratio 95% Confidence Limits** | | | | **P-value** | |
| Age (75+ as a reference category) | | | |  | | |  | |  | |  | |
| 18-64 | | | | 1.33 | | | 1.29 | | 1.38 | | <0.001 | |
| 65-74 | | | | 0.99 | | | 0.96 | | 1.03 | | 0.606 | |
| Male | | | | 1.02 | | | 0.99 | | 1.05 | | 0.246 | |
| Embolic or Primary Ischemic Stroke | | | | 0.96 | | | 0.88 | | 1.05 | | 0.370 | |
| Dyspepsia or Stomach Discomfort | | | | 1.10 | | | 1.06 | | 1.14 | | <0.001 | |
| Congestive Heart Failure | | | | 0.95 | | | 0.92 | | 0.98 | | 0.003 | |
| Coronary Artery Disease | | | | 1.02 | | | 0.98 | | 1.05 | | 0.353 | |
| Diabetes | | | | 0.88 | | | 0.86 | | 0.91 | | <0.001 | |
| Hypertension | | | | 0.90 | | | 0.87 | | 0.93 | | <0.001 | |
| Renal Disease | | | | 1.03 | | | 0.99 | | 1.08 | | 0.141 | |
| Myocardial Infarction | | | | 1.05 | | | 1.00 | | 1.11 | | 0.066 | |
| History of Stroke or TIA | | | | 0.86 | | | 0.79 | | 0.92 | | <0.001 | |
| History of Bleeding | | | | 1.12 | | | 1.08 | | 1.16 | | <0.001 | |

TIA: Transient Ischemic Attack

*Adjusted hazard ratios were estimated from a Cox proportional hazards model with factors for age, sex, embolic or primary ischemic stroke, dyspepsia or stomach discomfort, congestive heart failure, coronary artery disease, diabetes, hypertension, renal disease, myocardial infarction, history of stroke or transient ischemic attack, and history of bleeding.
